# YEAR ONE AND BEYOND: SUSTAINING A COLLABORATIVE AGING FELLOWSHIP PROGRAM

**DOI:** 10.1093/geroni/igac059.727

**Published:** 2022-12-20

**Authors:** Diane Martin

**Affiliations:** University of Maryland, Baltimore Graduate School, Baltimore, Maryland, United States

## Abstract

A graduate aging fellowship experience can provide participants with unique opportunities for professional development in interdisciplinary and transdisciplinary research, community engagement, and team science. Program success is contingent on multiple factors beyond participant recruitment and customizable curriculum to meet the educational goals of individual participants. Stakeholder buy-in, strategic planning, and identification of funding streams are all necessary to support an internal program beyond its first year. In this session, we will offer guidance in planning, building, and sustaining a fellowship program for current graduate students with a professional interest in supporting older adults.

